# The optimal induction dose of ciprofol combined with low-dose rocuronium in children undergoing daytime adenotonsillectomy

**DOI:** 10.1038/s41598-023-49778-8

**Published:** 2023-12-14

**Authors:** Dongjie Pei, Li Zeng, Ting Xiao, Lei Wu, Lei Wang, Siwei Wei, Zhen Du, Shuangquan Qu

**Affiliations:** Department of Anesthesiology, Hunan Children’s Hospital, 86 Ziyuan Road, Yuhua District, Changsha, 410000 Hunan China

**Keywords:** Medical research, Paediatric research, Clinical trial design

## Abstract

Adenotonsillectomy is the most common daytime surgery performed on children. Anesthesiologists must select the optimal combination of drugs to ensure effective anesthesia effect and prompt recovery in children. The optimal induction dose of ciprofol in children is unclear. In this study, we aim to investigate the effect of different doses of ciprofol on anesthesia induction in children undergoing daytime adenotonsillectomy and provide a reference for clinical use. 144 children aged 3–12 years, ASA I-II, undergoing daytime adenotonsillectomy, were included in this clinical trial. The children were randomly divided into three groups and given 0.4 mg/kg (C4), 0.6 mg/kg (C6), or 0.8 mg/kg (C8) of ciprofol for anesthesia induction. The primary outcome was intubation conditions. Vital signs and injection pain were also recorded. The rates of unacceptable intubation conditions were 30.6%, 8.7%, and 8.2% in the C4, C6, and C8 groups (*P* value < 0.0167). The overall incidence of reported injection pain was 3.5%. The heart rate and mean arterial pressure did not differ between the groups at the same time points. We found that combining 0.6 mg/kg of ciprofol with low-dose rocuronium could provide optimal intubation conditions in pediatric daytime adenotonsillectomy patients. This combination resulted in stable circulation and BIS values. This study is registered at the Chinese Clinical Trial Registry (Registration number: ChiCTR2200063144, Date of Registration: 31/08/2022)

## Introduction

Anesthesiologists face greater challenges when administering anesthesia to children during daytime surgeries. It requires careful selection of medications and their optimal combination to ensure effective anesthesia and prompt recovery of the child.

Adenotonsillectomy is the most common daytime surgery for children. Anesthesiologists typically use tracheal intubation to ensure the safety of the airway during the procedure. Neuromuscular blockers (NMBs) are commonly used to facilitate smooth intubation. In certain elective surgeries, anesthesiologists may try to perform intubation using propofol and opioids without NMBs. However, this approach can pose challenges in achieving optimal intubation conditions^1,2^. However, while conventional doses or even higher doses of NMBs can achieve satisfactory intubation conditions, such doses may delay awakening or result in residual neuromuscular blockade^3^. Some studies have found that the use of low-dose NMBs in combination with other general anesthetics can help reduce the stress response caused by tracheal intubation. This combination also contributes to achieving satisfactory intubation conditions in children without affecting their extubation and awakening time^4–7^. However, there are still differences in intubation conditions among these studies, indicating that intubation conditions are influenced not only by NMBs but also by other anesthetic drugs.

Ciprofol is a novel general anesthetic that exhibits rapid onset and recovery times comparable to propofol. It also has low incidences of injection pain and respiratory depression^8^. These advantages may make it suitable for use in pediatric daytime surgery anesthesia. The influence of induction doses of cipofol combined with low-dose rocuronium on intubation conditions in children has not been studied yet. In this prospective, single-center, double-blinded, randomized study, we compared intubation conditions and hemodynamic changes associated with three doses of ciprofol. Our aim was to investigate the optimal induction dose of ciprofol when combined with low-dose rocuronium in pediatric adenotonsillectomy.

## Materials and methods

This study was approved by the Medical Ethics Committee of Hunan Children’s Hospital (HCHLL-2022–079) and registered with the Chinese Clinical Trial Registry (Registration number: ChiCTR2200063144, Date of Registration: 31/08/2022). All experiments were conducted in compliance with the Declaration of Helsinki and relevant guidelines.

Children aged 3–12 years, who had a normal preoperative examination, no history of surgery or trauma, BMI ≤ 20, and an ASA class I–II, were selected to sign an informed consent form prior to undergoing daytime adenotonsillectomy at our hospital. The exclusion criteria included a history of traumatic surgery or allergies, abnormal preoperative findings, comorbid inherited metabolic diseases, and altered surgical procedures.

Children were randomly divided into the C4, C6, and C8 groups, and were respectively injected with ciprofol at doses of 0.4 mg/kg, 0.6 mg/kg, and 0.8 mg/kg during the induction. Our study employed double-blinding. After the parents of the children have signed the informed consent form, the investigator will open their randomization envelope and assign the child to the corresponding group. At the same time, the investigator handed the pre-prepared induction medication to the anesthesiologist in charge of the surgery. The medication, ciprofol, is contained in a syringe with an obscured barrel, making the volume of the medication not visible to the anesthesiologist. Assessment of endotracheal intubation and intubation conditions was conducted by an experienced anesthetist with over 5 years of expertise in pediatric anesthesia. This individual was also responsible for documenting vital signs. Children, their parents, and anesthesiologists were blinded to group allocation.

### Anesthesia process

Children were admitted to the operating room with a peripheral intravenous catheter. Their heart rate (HR), mean arterial pressure (MAP), oxygen saturation, and bispectral index (BIS) were monitored. The selected dose of ciprofol was injected during anesthesia induction, and the injection took 30 s. Children were asked if they experienced any injection pain or other discomfort. Afterward, they were administered sufentanil 0.4 mcg/kg and rocuronium 0.3 mg/kg.

Endotracheal intubation was performed 90 s after the injection of rocuronium. Intubation conditions were also evaluated. Ciprofol 0.2 mg/kg was administered when BIS was above 75 prior to tracheal intubation (T3). When the intubation conditions were unacceptable, 0.3 mg/kg of rocuronium was administered to ensure successful intubation.

Anesthesia was maintained with sevoflurane 2.5–3% (inspired concentration), which was adjusted based on the patient’s vital signs. Sevoflurane inhalation was discontinued at the end of the procedure, and the child was transferred to the postanesthesia care unit (PACU). Extubation and awakening were completed in the PACU, and sugammadex 2 mg/kg was administered to reverse the effect of rocuronium. The child was extubated when spontaneous breathing returned and end-tidal CO_2_ was at 40–45 cmH_2_O. Children were returned to the ward when the Aldrete score was 9 or higher.

The primary outcome was intubation conditions, and the secondary outcomes included vital signs and injection pain. The HR, MAP, and BIS values of the children were recorded at different time points: before induction (T1), 30 s after ciprofol injection (T2), before intubation (T3), 1 min after intubation (T4), and 5 min after intubation (T5). Intubation conditions were assessed as described in previous literature (Table 1)^9^.

**Table 1:** Assessment of intubation conditions.

Assessment of intubation conditions			
Variables	Intubation conditions		
	Clinically acceptable		Clinically not acceptable
	Excellent	Good	Poor
Laryngoscopy	Easy	Fair	Difficult
Vocal cords			
*Position*	Abducted	Intermediate	Closed
*Movement*	None	Moving	Closing
Reaction to insertion of tracheal tube and/or cuff inflation			
*Movement of the limbs*	None	Slight	Vigorous
*Coughing*	None	Diaphragm	Sustained > 10 s

The overall intubation condition was assessed according to the circumstances of each variable: if all variables were rated as excellent or good, the intubation condition was clinically acceptable, and if either variable was assessed as poor, the intubation condition was clinically not acceptable.

### Statistical analysis

The sample size was calculated by 90% power of the study. Fifty children per group were required to determine a minimal allowable difference of 15% in clinically unacceptable intubation conditions between groups by an alpha error of 0.05. Comparisons between groups for intubation conditions in children were performed using the χ^2^ test or Fisher’s exact test. Demographics and vital signs were determined by $\overline{x }\pm s$, and one-way ANOVA was used for inter- and intragroup comparisons. Statistical analysis was performed using SPSS (v19.0).

## Results

**Figure 1:**
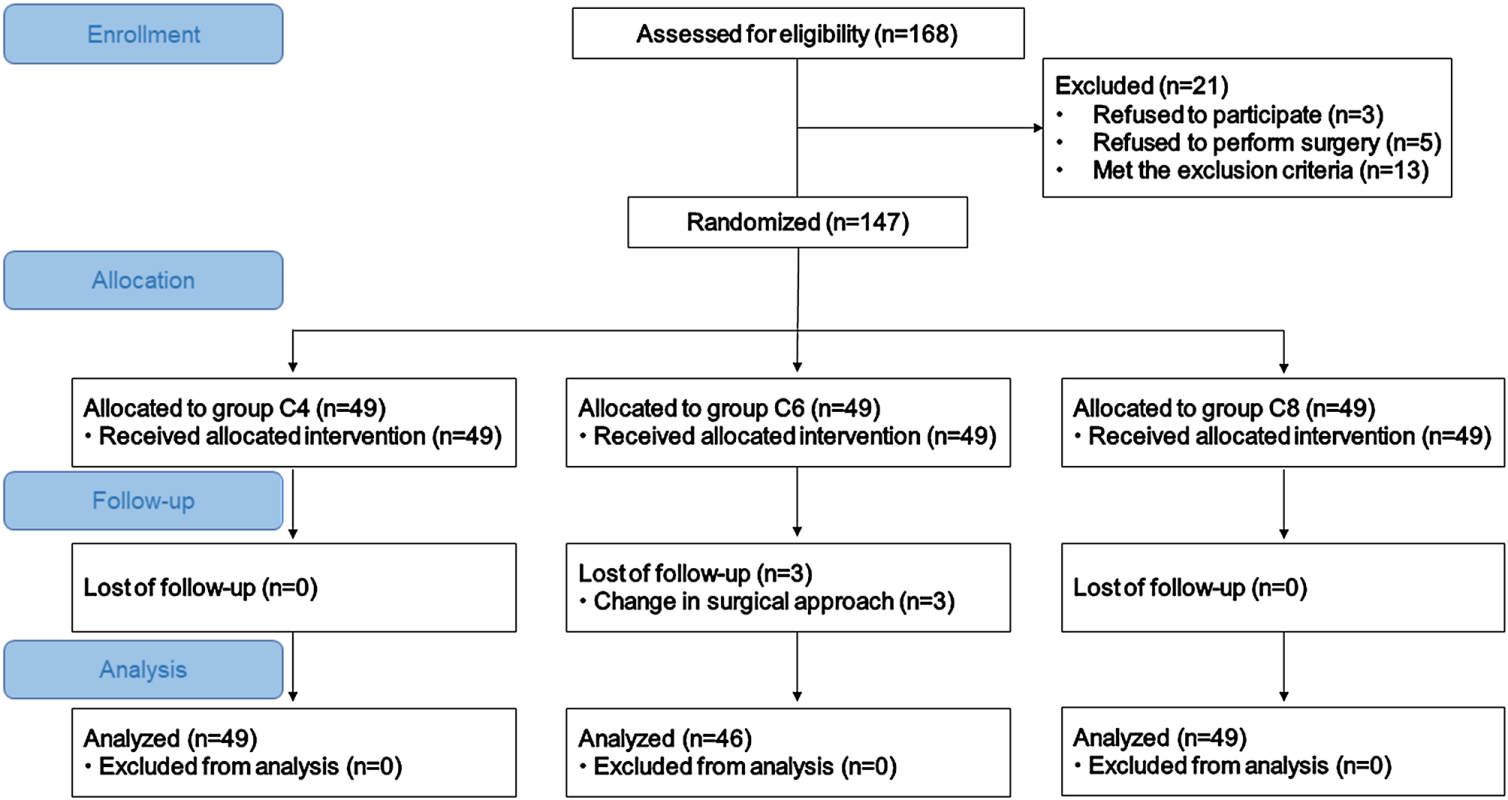
Flow chart. Group C4: ciprofol dose 0.4 mg/kg. Group C6: ciprofol dose 0.6 mg/kg. Group C8: ciprofol dose 0.8 mg/kg.

A total of 168 children were screened, and 147 were enrolled. Patients were excluded for the following reasons: exclusion criteria (n = 13), refusal of surgical treatment (n = 5), and refusal to participate in the study (n = 3). Three children in group C6 were excluded due to changes in surgical procedures. Overall, 144 children completed the study, specifically, 49 in group C4, 46 in group C6, and 49 in group C8. The flow chart is shown in Fig. 1.

There were no statistically significant differences in the demographics of the children or the anesthesia and operation times (Table 2).

**Table 2:** Comparison of general conditions and anesthesia/operative times.

	C4	C6	C8	*P* value
Age (year)	5.58 ± 1.79	5.89 ± 1.88	5.68 ± 2.13	0.738
Sex (M/F)	30/19	32/14	28/21	0.484
BMI (m/kg^2^)	15.25 ± 1.72	15.45 ± 2.39	15.28 ± 1.80	0.861
Anesthesia time (min)	36.08 ± 16.00	33.57 ± 8.97	37.61 ± 10.99	0.281
Operative time (min)	25.57 ± 16.53	23.04 ± 8.62	26.63 ± 10.80	0.362

*BMI* body mass index.

**Figure 2:**
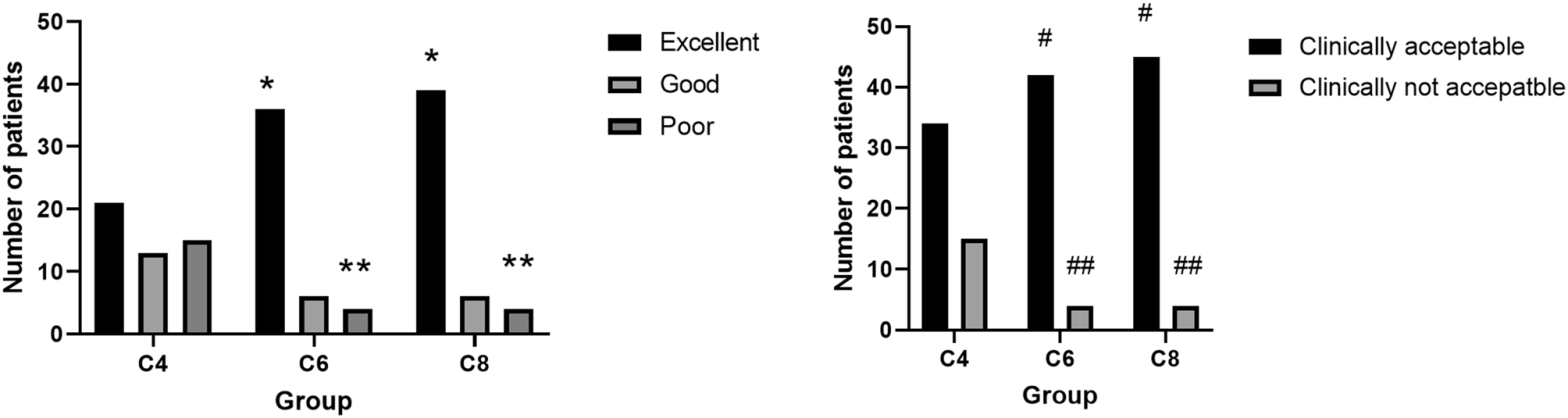
Intubation conditions of the three groups (*, **, #, ## there was a significant difference compared with Group C4).

The rates of intubation conditions deemed clinically unacceptable were 30.6%, 8.7%, and 8.2% in the three groups, with statistically significant differences observed between the groups (*P* value < 0.0167). Compared to the C4 group, the C6 and C8 groups had significantly lower rates of unacceptable intubation conditions. The percentages of excellent, good, and poor results in each group were as follows: 42.9%, 26.5%, and 30.6% in group C4; 78.3%, 13.0%, and 8.7% in group C6; and 79.6%, 12.2%, and 8.2% in group C8, respectively (Fig. 2, Table 3).

**Table 3:** Comparison of intubation conditions among the three groups.

Variable	C4	C6	C8	*P* value
Intubation conditions				
Clinically acceptable/Clinically not acceptable	34/15	42/4	45/4	0.005
Excellent/Good/Poor	21/13/15	36/6/4	39/6/4	0.001
Laryngoscopy				
Clinically acceptable/Clinically not acceptable	49/0	46/0	49/0	
Easy/Fair/Difficult	49/0/0	46/0/0	49/0/0	
Vocal cords position				
Clinically acceptable/Clinically not acceptable	49/0	46/0	49/0	
Abducted/intermediate/closed	49/0/0	45/1/0	49/0/0	0.319
Vocal cords movement				
Clinically acceptable/Clinically not acceptable	49/0	46/0	49/0	
None/moving/closing	49/0/0	45/1/0	49/0/0	0.319
Movement of the limbs				
Clinically acceptable/Clinically not acceptable	38/11	44/2	47/2	0.007
None/slight/vigorous	24/14/11	37/7/2	40/7/2	0.002
Coughing				
Clinically acceptable/Clinically not acceptable	45/4	44/2	47/2	0.732
None/diaphragm/sustained	41/4/4	42/2/2	46/1/2	0.574

HR and MAP at each time point were not significantly different between the groups. BIS was significantly different between the groups at all time points except T1 and T5 (Table 4). Among the different time points, there were significant differences in pairwise contrasts at T2. At T3, the C4 BIS was significantly higher than the C6 and C8 values at T3. At T4, the C4 BIS was significantly higher than the C8 value at T4. Lastly, at T5, the C8 BIS was significantly lower than the C4 and C6 values.

**Table 4:** Between-group comparisons of vital signs.

	C4	C6	C8	*P* value
HR bpm				
T_1_	94.12 ± 19.36	96.22 ± 17.99	94.39 ± 17.57	0.834
T_2_	97.61 ± 21.35	98.30 ± 13.95	100.27 ± 16.52	0.743
T_3_	110.29 ± 16.40	113.67 ± 12.05	110.53 ± 13.31	0.431
T_4_	117.73 ± 12.62	120.98 ± 15.63	118.88 ± 15.96	0.559
T_5_	113.73 ± 14.23	114.59 ± 14.56	113.18 ± 14.99	0.895
MAP mmHg				
T_1_	79.16 ± 9.75	82.00 ± 9.96	78.92 ± 9.07	0.227
T_2_	80.59 ± 11.59	79.13 ± 10.75	77.69 ± 9.89	0.414
T_3_	88.73 ± 10.88	88.57 ± 12.54	85.71 ± 9.50	0.318
T_4_	94.37 ± 12.02	90.54 ± 10.97	91.61 ± 13.89	0.299
T_5_	82.67 ± 10.51	81.96 ± 11.55	81.04 ± 11.82	0.774
BIS				
T_1_	95.53 ± 2.85	94.91 ± 3.33	95.35 ± 2.46	0.569
T_2_	56.82 ± 11.51	46.98 ± 10.37	39.82 ± 11.46	0.000
T_3_	60.43 ± 8.12	56.26 ± 7.17	55.33 ± 6.80	0.002
T_4_	63.10 ± 6.62	61.15 ± 6.51	59.63 ± 6.87	0.039
T_5_	60.92 ± 5.82	60.41 ± 6.50	57.98 ± 6.42	0.053

*HR* heart rate, *MAP* mean arterial pressure, *BIS* bispectral index.

**Figure 3:**
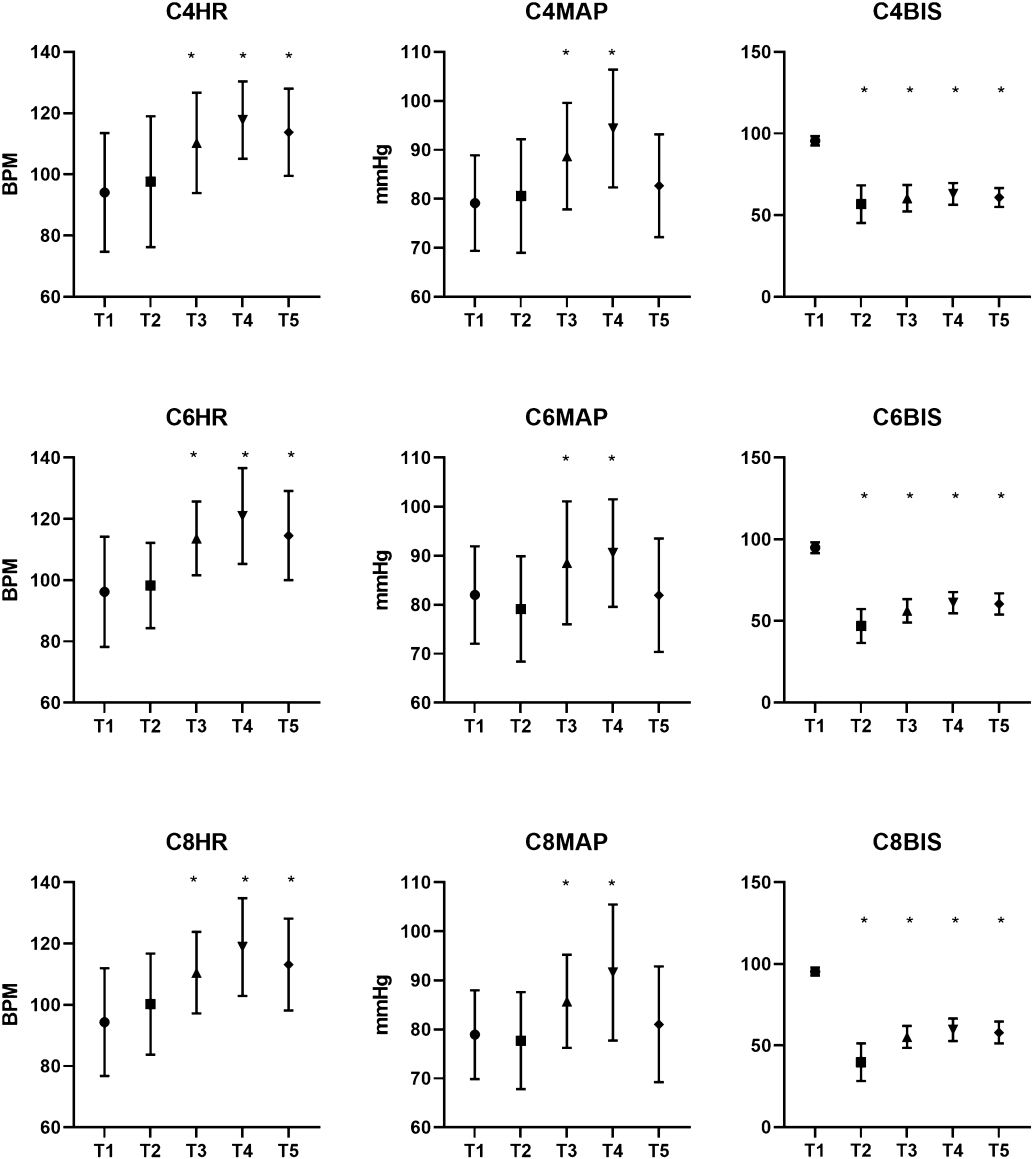
Within-group vital sign comparisons (* there was a significant difference compared with T1).

Comparisons of vital signs within groups are shown in Fig. 3. The statistical results indicated that the HR in each group was significantly higher at T3, T4, and T5 compared to T1. Additionally, the MAP was significantly higher at T3 and T4 in each group before returning to preinduction levels. The BIS values were significantly lower at T2, T3, T4, and T5 compared to preinduction levels.

No children were given an additional dose of ciprofol because none had a BIS greater than 75 at T3. A total of 23 children with poor intubation conditions were re-injected with rocuronium to complete intubation. This included 15 children in the C4 group and 4 children each in the C6 and C8 groups.

Minor injection pain was observed in 5 out of the 144 children, and its incidence was approximately 3.5%. No other adverse effects were reported.

## Discussion

Anesthesia for day surgery in pediatric patients is more challenging than in adults due to physiological peculiarities. Adenotonsillectomy is a frequently performed pediatric day surgery procedure that often necessitates endotracheal intubation to ensure the child’s airway secured. Neuromuscular blockers (NMBs) are typically administered to facilitate intubation. To achieve this, high doses of NMBs are used for a rapid onset time^10^. However, the duration of action can be prolonged and may even cause residual neuromuscular block^11,12^. This can make it difficult to ensure postoperative safety for pediatric patients. Although endotracheal intubation can be accomplished without the use of NMBs, it require high doses of opioids to achieve suitable intubation conditions, which can result in delayed awakening^13^. As a result, this method is rarely used in children. It has been shown that acceptable intubation conditions can be achieved with a low dose of rocuronium during short-term surgery. However, high-dose rocuronium prolongs muscle recovery without improving intubation conditions. Furthermore, the increased use of NMBs may be linked to readmission within 30 days after surgery^14,15^. Intubation conditions are not only related to NMBs but also associated with induction drugs^16,17^. Therefore, a low dose of NMBs combined with other induction drugs can be used to achieve optimal intubation conditions and ensure safety during daytime adenotonsillectomy in children.

Propofol is currently the most commonly used anesthetic in clinical settings. It is known for its effectiveness in including rapid awakening and is often the preferred choice for pediatric day surgery. However, one of the most common adverse effects of propofol is injection pain, which affects approximately 25–85% of children^18^. Injection pain can significantly impact the patient’s experience, leading to increased tension and anxiety. It can also affect the effectiveness of anesthesia induction^8^. Ciprofol is a novel general anesthetic that acts as a short-acting agonist on the gamma-aminobutyric acid-A (GABAA) receptor. It enhances chloride influx mediated by the GABAA receptor, resulting in sedative and anesthetic effects. Ciprofol has a receptor affinity and potency of approximately five times higher than propofol^8,19^. The main metabolic pathways of ciprofol are oxidation, glucuronidation, and sulfation. The metabolites are primarily excreted in the urine, with an elimination half-life of 90 min^20^. The potential side effects of ciprofol include hypoxemia, bradycardia, hypotension, and injection pain^21,22^.

The overall incidence of injection pain for ciprofol in our study was 3.5%, which was much lower than the incidence of propofol injection pain reported^23,24^. This may be because of the higher lipid solubility and low concentration of the dissociated drug in the aqueous phase under equivalent conditions, which effectively reduces the occurrence of injection pain^22^. This suggests that ciprofol may enhance the stability of the anesthesia induction process and improve the overall experience for children.

Propofol causes a dose-dependent decrease in blood pressure and HR during induction^25,26^, which can be attributed to the inhibition of cardiac muscle contraction or vascular tone^27,28^. This effect is particularly harmful for pediatric patients. In previous clinical studies of ciprofol, researchers reported similar effects on HR and blood pressure, which led to temporary suppression of circulatory function^8^. Based on our results, ciprofol at our study dose had a lesser impact on the circulatory system in children during the induction phase. We observed a significant increase in HR and an increase in MAP that persisted until the end of induction intubation (T4). The phenomenon of increased HR after ciprofol injection was also observed in healthy subjects at Soochow University^20^. This may be related to the high potency of ciprofol and the mild inhibition of circulatory function^29^. However, since previous studies were conducted in adults, it is unclear whether this result is associated with the robust cardiovascular compensatory capacity of pediatric patients.

BIS is correlated with clinical indicators of anesthesia. As the depth of anesthesia increases, BIS diminishes^30^. Previous researchers have found that ciprofol has a similar effect on BIS as propofol^31,32^. In our study, we found that the decrease in BIS was related to the dose of ciprofol. Furthermore, the larger the dose, the more pronounced the decrease. These differences between groups were only limited to the initial minutes after drug injection. However, 5 min after intubation, the BIS of each group gradually stabilized at approximately 60. This may be related to the rapid redistribution of the drug after a single injection of ciprofol^19^.

Sufentanil at a dose of 0.4 mcg/kg was administered for induction, taking into account the estimated duration of the operation (approximately 30 min). It was not planned to administer any additional doses of sufentanil during the operation. Studies have shown that it would counteract the cardiovascular responses conferred by intubation only when sufentanil amounts are ≥ 0.3 mcg/kg^33–35^. This facilitates our observation of the effects of ciprofol on the cardiovascular system during the induction phase. Because the wounds from daytime adenotonsillectomy are relatively large and the children require sufficient analgesia, an insufficient dose of sufentanil dose may result in pain or emergence agitation^36,37^. On the other hand, a large dose can cause delayed awakening. Therefore, we selected this particular dose.

Although rocuronium 0.6 mg/kg (2 × ED95) has a rapid onset of action and provides satisfactory intubation conditions^6^, it may cause residual neuromuscular blockade for the short surgical procedure due to its long recovery time of 42–82 min^38,39^. When low doses of medium-acting NMBs are used, their effects become short-acting^40^. Thus, low-dose rocuronium may be appropriate for children undergoing short-duration surgery. In this study, we referred to previous reports and chose a low dose of 0.3 mg/kg rocuronium for induction^5,41,42^. The onset time for NMBs was determined by monitoring the time from injection to maximal T1 block in the adductor hallucis. However, it has been reported that neuromuscular blockades of the laryngeal adductor, diaphragm, masseter, etc., which are closely related to intubation conditions, have a more rapid onset than that of the adductor pollicis^43,44^. In addition, intubation conditions are correlated with the quality of anesthesia induction and the extent of drug-induced suppression of the laryngeal reflex^16,25^. Thus, complete paralysis of the adductor pollicis is not a reliable indicator for assessing intubation time and conditions^45–47^. Therefore, we did not use neuromuscular block monitoring to determine the onset time of rocuronium in this study. Instead, we relied on the previously established onset time of 87 s (90 s) for 0.3 mg/kg (ED95) rocuronium as the time for intubation and assessment^6^.

Our results suggest that the rate of unacceptable intubation conditions in the C4 group was much higher than that of the other two groups, indicating that the induction dose of 0.4 mg/kg ciprofol was too low for pediatric patients. Notably, the recommended starting dose for adult induction is 0.4 mg/kg. This difference may be attributed to a larger extracellular fluid volume in children. When we increased the induction dose to 0.6 mg/kg, the rate of intubation conditions that were clinically acceptable significantly increased. Increasing the ciprofol dose to 0.8 mg/kg did not further improve intubation conditions, suggesting that the anesthetic effect of ciprofol may have reached a plateau. As observed in the specific parameters for assessing intubation conditions, the primary difference among the three groups was predominantly in limb movement. This difference was most pronounced at the low dose, possibly due to fasciculation. There is a possibility that fasciculations, which are caused by the inhibition of gamma-aminobutyric acid and result in increased sensitizes of the cortex, can lead to hyperexcitability in response to even small amounts of stimulation. This condition is mostly associated with low doses of anesthetic drugs, as most fasciculations occur at low drug concentrations during the induction and awakening periods^18^. Therefore, for day surgery in children, an induction dose of less than 0.6 mg/kg of ciprofol may cause involuntary movements that could have a negative impact on intubation.

Based on these findings, we conclude that a combination of 0.6 mg/kg ciprofol and low-dose rocuronium can provide satisfactory intubation conditions in children undergoing daytime adenotonsillectomy. This combination also helps maintain circulatory stability and ensures prompt awakening.

Our study had some weaknesses. Aspects of vital signs were only observed during the induction phase. It was not whether ciprofol can affect circulatory function during surgery. In addition, we only observed the overall incidence of injection pain and did not investigate whether it was correlated with the dosage of ciprofol. Last, we did not record whether the children experienced agitation upon awakening after extubation, which is an important factor in evaluating the effectiveness of the medication.

## Conclusion

A combination of 0.6 mg/kg ciprofol and a low dose of rocuronium can provide satisfactory tracheal intubation conditions and ensure stable circulation and BIS in children undergoing daytime adenotonsillectomy.

## Data Availability

The data that support the findings of this study are available from the corresponding author Shuangquan Qu upon reasonable request.
